# Evaluation of the Efficacy of a Digital Chest Drainage System in Traumatic Pneumothorax

**DOI:** 10.7759/cureus.41188

**Published:** 2023-06-30

**Authors:** Shiva Thakur, Nitin Kumar Kashyap, Gaind Saurabh, Pranay Mehsare

**Affiliations:** 1 Cardiothoracic Surgery, All India Institute of Medical Sciences, Raipur, Raipur, IND

**Keywords:** air leak, digital drainage system, thopaz, chest trauma, pneumothorax

## Abstract

Introduction: Pneumothorax is the major complication in patients with chest trauma. Thoracic injury is a major cause of trauma-related deaths, with up to half of these patients developing pneumothorax. The initial primary management of pneumothorax is intercostal chest drainage (ICD). Chest drainage systems are used to resolve pleural air leakage (PAL), lymphatic or exudative effusion, blood accumulation after chest surgery or trauma, and other disease conditions such as pneumothorax. This study evaluates the efficacy of a digital chest drainage system (Thopaz^+^, Medela AG, Baar, Switzerland) in patients with pneumothorax following chest trauma and analyzes the satisfaction score by patients.

Method: A hospital-based cross-sectional study was conducted in a tertiary care centre at the Department of Cardiovascular and Thoracic Surgery (CTVS). All patients with a diagnosis of traumatic pneumothorax/hemopneumothorax from January 2021 to June 2022, aged more than 15 years, were enrolled for the study. A total of 102 patients required chest drainage systems and were selected for the study. We analysed demographic data, clinical profiles, and routine investigations with chest X-rays and computed tomography (CT) scans. All patients were connected with digital drainage devices and monitored for air leaks and other complications. Patient satisfaction was evaluated by a purposefully developed survey questionnaire.

Results: Most of our study subjects were male (84.3%) and the mean age was 42.38±15.75 years. The total duration of chest tube, post-operative air leak and duration of hospital stay were noted. The mean chest tube duration was 4.39±1.18 days. Twelve patients were found to have air leaks with digital drainage devices. The mean duration of hospital stay was 5.75±1.49 days. All subjects were provided with a survey questionnaire to assess their response to digital drainage devices. We found that patients were comfortable and had positive responses for the Thopaz^+^ device.

Conclusion: We found that Thopaz^+^ digital drainage system is useful in reducing chest tube duration and hospital stay. It also helps in the early resolution of air leaks and minimises complications. Most of our patients showed a positive attitude. With regard to Thopaz^+^ digital device, our study concludes that Thopaz^+^ should be considered for patients who need chest tube drain for pneumothorax.

## Introduction

Trauma is the most dreaded emergency in current society. The rapid increase in the number of automobiles along with industrialization results in vehicular accidents, causing an increased incidence of trauma [1]. Chest injury is potentially the most serious emergency of all; hence, its management should be done as early as possible [2]. Chest injuries account for 20-25% of deaths due to trauma. In India, approximately 16,000 deaths happen every year due to thoracic injury [3]. The main complication of chest trauma is the collection of air in the chest cavity leading to a shift of mediastinum, causing a life-threatening emergency [4].

Pneumothorax is the collection of air inside the pleural cavity (between the visceral and parietal pleura) which separates the lung from the chest wall. Depending upon the aetiology, this can be classified into traumatic, iatrogenic, primary, or secondary. Traumatic pneumothorax can be the result of blunt or penetrating chest trauma. Blunt trauma can cause rib fracture, increased thoracic pressure, and bronchial injury [4]. Penetrating injuries (stab wounds and gunshot wounds) mainly injure the peripheral lung, producing both pneumothorax and haemothorax in the majority (more than 80%) of all penetrating chest wall injuries [4].

An inter-costal chest drain is the most definitive primary management of the pneumothorax [5]. A digital drainage system comprises water seal bottle and negative pressure suction device. Continuous controlled negative pressure suction is very effective for air evacuation and healing of the pleural fistula. It also avoids chest tube (inter-costal tube) obstruction due to adhesion or fibrous bands in the pleura. A negative pressure suction device should be considered in the presence of continuous air leakage or incomplete lung expansion. At present, it is available as a high-volume low-pressure suction device with a general pressure regulator range between 5-20 cm H2O [6].

Traditional chest drainage systems have been found inconvenient to patients and hospital staff as these systems are unable to provide accurate data on drain output and air leakage. Difficulty in mobility and patient discomfort are the main disadvantages of traditional systems. Traditional chest drainage increases the chance of infection whenever it is disconnected for patient mobilization. Traditionally, to achieve continuous negative suction, chest drain bottles are connected to low-pressure wall suction. The wall suction is extremely unreliable and variable. Traditional systems don’t have the facility to record the air leak amount objectively and accurately. Traditional water-based systems allow monitoring of air leaks by visualization and assessment of bubbles in a water bag. This leads to inter-observer variability and false interpretation of air leaks [7].

Thopaz^+^(Medela AG, Baar, Switzerland) is a newly developed electronic chest drainage device. Thopaz^+^ is a portable negative suction device having a canister (for drainage) along with a charger and allows easy and early mobilization of the patient. It maintains controlled negative suction pressure constantly as pre-set by the user. The amount of fluid collected due to haemothorax and air leak is measured with a calibrated container. This helps the medical team to plan further management depending upon recorded data, unlike in traditional drainage systems where data recording advantages are not present [7].

Digital devices have various alarms for different situations like tube obstruction, tube disconnection, and suction failure. They also provide better safety for both medical healthcare personnel and patients as the drainage fluid has no communication with the outer environment due to it being a closed system. Along with this, such devices remove inter-observer variability as digital measurements of air leaks are recorded (ml/minute) and displayed on the device screen [8]. According to the manufacturer, Thopaz^+^ decreases hospital stay, allows for cost savings, increases convenience for medical personnel, improves drain management, and has better patient outcomes [9].

Pneumothorax is an urgent situation and should be treated immediately upon diagnosis. Chest tube placement not only helps the lung to expand but also removes the collection inside the pleural cavity. A digital drainage system enables expansion of the lung, complete evacuation of the collection, and pleural fistula healing if present. Such a device provides suction only when needed until the pleural pressure crosses the physiological limit. It allows the patients to mobilise easily and records the flows of the drainage in a digitally retrievable format, which is not possible with traditional drainage systems. In previous studies, it has been found that patients managed with electronic devices had a more positive perception of the chest drainage system, particularly related to its comfort, portability, and convenience, compared with those managed with the traditional device [10].

There is very little data available on using a digital drainage device in traumatic pneumothorax in the Indian population. This study would help to define the role of digital drainage systems in managing traumatic pneumothorax.

## Materials and methods

This prospective observational study was conducted in the Department of Cardiovascular and Thoracic Surgery (CVTS), at All India Institute of Medical Sciences (AIIMS), Raipur, India, from January 2021 to June 2022. The study was approved by the Institutional Ethics Committee, AIIMS, Raipur, India (approval number: 1486/IEC-AIIMSRPR/2021, dated February 16, 2021). All patients (both male and female), aged more than 15 years, who were diagnosed with traumatic pneumothorax/hemopneumothorax requiring chest tube insertion were included in the study. Patients with pre-existing coagulopathy (international normalized ratio (INR) > 2) or platelet count < 50,000/µL were excluded.

**Figure 1:**
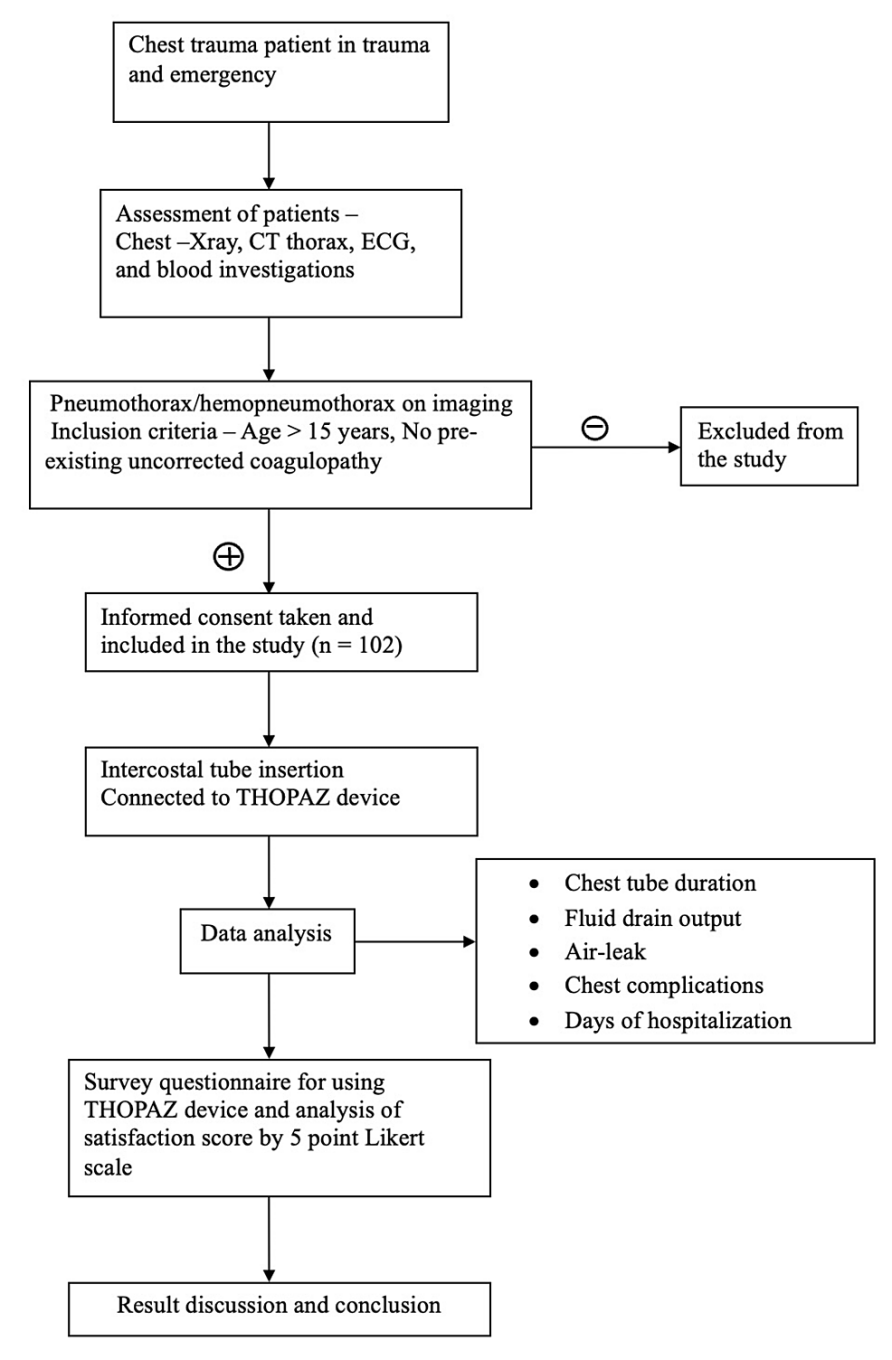
Flow chart showing study design Thopaz^+ ^device, Medela AG, Baar, Switzerland

A total of 102 patients were enrolled. Data collection was performed prospectively at the time of the study. Demographic information (age, sex, residence), presenting symptoms (chief complaints, duration, and severity), associated complaints/morbidities, past history of tuberculosis, asthma and lung disease, and aetiology of chest trauma were recorded at entry into the study. General and systemic examinations were noted. Routine blood investigation, electrocardiogram, and reports of X-ray and CT of the thorax were entered into a specific proforma designed for the study. Figure 1 shows the flow chart of the study design.

**Figure 2:**
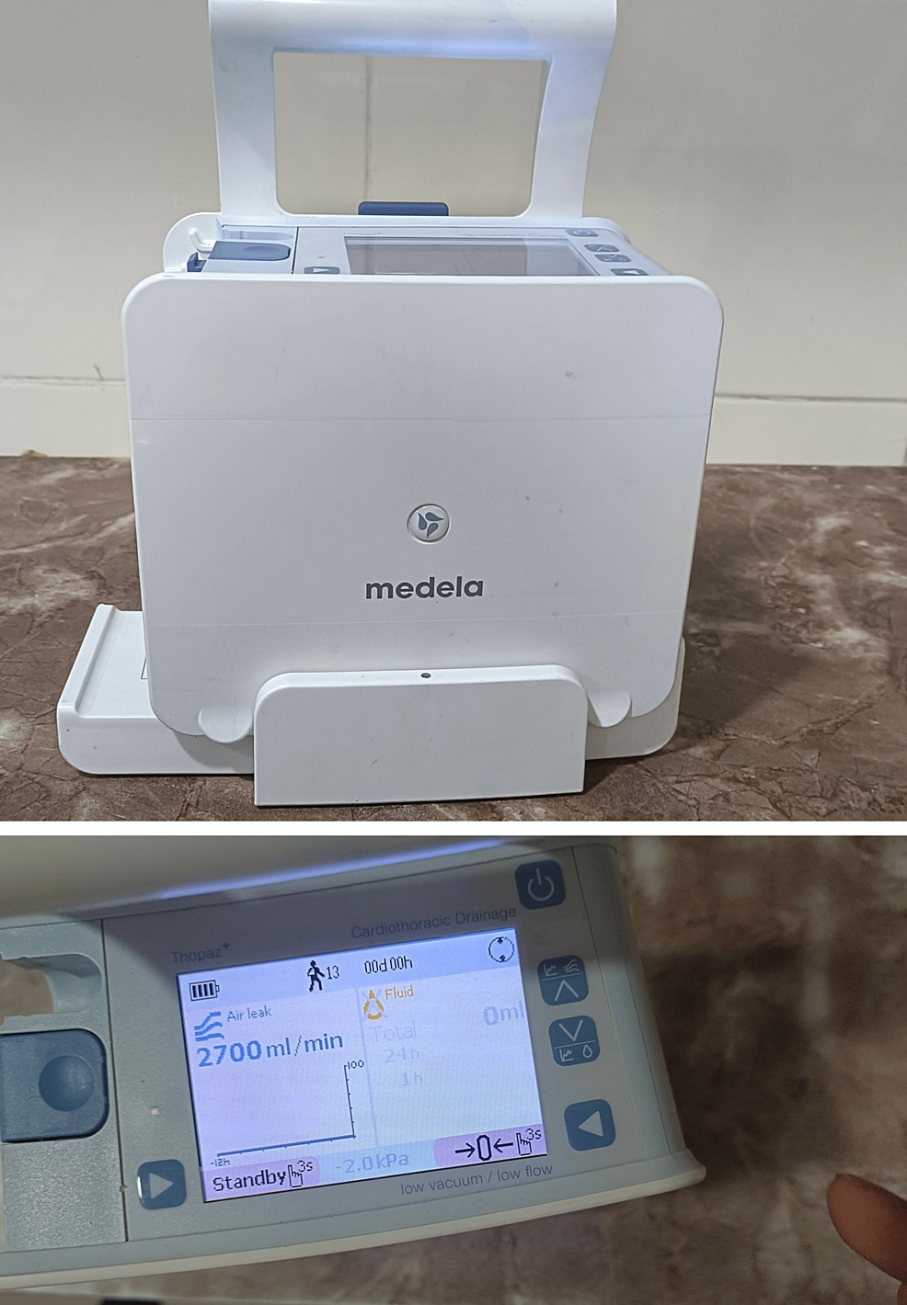
Thopaz+ digital drainage with display Medela AG, Baar, Switzerland

Informed consent was obtained preoperatively from patients with pneumothorax and requiring chest tube insertion, before chest tube insertion and application of digital chest drainage system. Patients connected with the digital drainage device (Figure 2) were managed by keeping the suction pump at -20 cm H2O up till the next post-operative day and then the suction pump was kept on physiological level (-8 cm H2O) thereafter. Chest tubes were removed when air leak was less than 30 mL/minute for at least eight hours in the absence of significant fluctuation of air leak on the graph, and when drain output was lesser than the standard parameter of our centre. Post-operative lung expansion, air leak, number of days of the chest tube, tube reinsertion, number of days of hospital stay, and chest complication were entered in the Excel chart (Microsoft Corporation, Redmond, Washington, United States) by the surgical and intensive care team.

Patient satisfaction with the use of the digital device was evaluated by a purposefully developed survey questionnaire (Table 1). It contains five multiple-choice questions measured on a five-point Likert-type scale.

**Table 1:** Survey questions and their relative scores on a five-point Likert-type scale used to assess satisfaction of patients with the chest drainage system

Questionnaire	Multiple Choice Answers and Scores
Question 1. Do you feel comfortable to move out of bed with chest drainage system?	1.Very Uncomfortable
	2. Uncomfortable
	3.Neither comfortable nor uncomfortable
	4.Comfortable
	5.Very comfortable
Question 2. How comfortable you feel while walking in room or ward with chest drainage system?	1.Very Uncomfortable
	2. Uncomfortable
	3. Neither comfortable nor uncomfortable
	4. Comfortable
	5.Very comfortable
Question 3. Do you think chest drainage system is convenient for other patients?	1.Very Inconvenient
	2. Inconvenient
	3.Neither convenient nor Inconvenient
	4.Convenient
	5.Very Convenient
Question 4. Are you comfortable in moving on bed or changing position at night with chest drainage system?	1.Very Uncomfortable
	2. Uncomfortable
	3.Neither comfortable nor uncomfortable
	4.Comfortable
	5.Very comfortable
Question 5. How safe you feel to move around with chest drainage system?	1.Very Difficult
	2. Difficult
	3.Neither easy nor Difficult
	4.Easy
	5.Very Easy

Data analysis

Data were entered into Microsoft Excel (windows 7, version 2007; Microsoft Corporation) and analyses were done using IBM SPSS Statistics for Windows, Version 22.0 (Released 2013; IBM Corp., Armonk, New York, India. Descriptive statistics such as mean and standard deviation for continuous variables, frequencies and percentages were calculated for categorical variables were determined. Bar charts and pie charts were used for visual representation of the analyzed data. The level of significance was set at 0.05.

## Results

A total of 102 subjects hospitalised at the CTVS department of AIIMS, Raipur, India, following chest trauma in the study period were included in the study. Of the 102 patients, the majority were in the age groups of 31-40 years and 41-50 years with 20 patients (19.6%) in each group. The next common age group was 21-30 years, with 19 (18.6%) patients. More than 50% of patients were in the third and fourth decades of life. The incidence of chest injury was low among very young and old patients. The mean age of presentation was 42.30 ± 15.88 years. There were 86 male and 16 female patients. The sex ratio was 5.3:1. 

The most common cause of chest trauma was road traffic accidents (RTA) (60.8%), followed by self-fall/blunt trauma (32.4%) and penetrating injury/stab injury (6.9%). The most common presenting symptom was chest pain (100%) followed by tachycardia (48%), tachypnoea (34.3%), dyspnoea (11.8%), and subcutaneous emphysema (11.8%). Ninety-two (90.2%) did not have any history of lung diseases and only 10 (9.8%) had past history of lung disease (asthma and tuberculosis).

**Figure 3:**
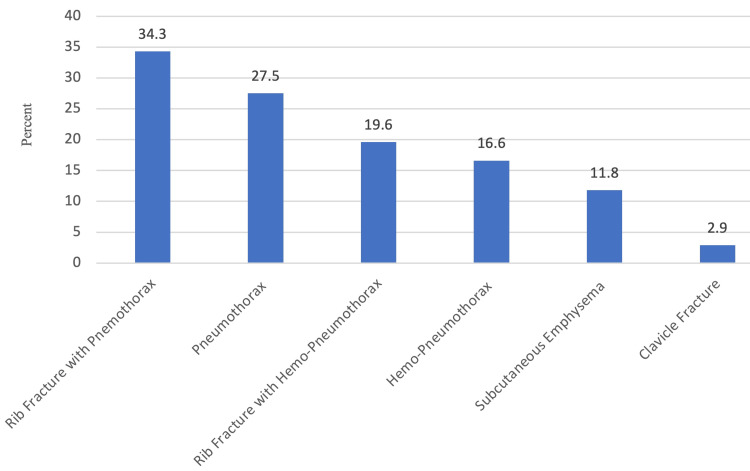
Distribution of chest X-ray findings

Of the 102 patients, 35 (34.3%) had findings of rib fracture with pneumothorax in chest x-rays; 28 (27.5%) patients had only pneumothorax in chest x-ray, 19 (18.6%) had rib fracture with hemopneumothorax, 16 (15.7%) had hemopneumothorax, 12 (11.8%) had subcutaneous emphysema while three (2.9%) had clavicle fracture in chest x-ray (Figure 3). Ninety-two (90.2%) patients had similar findings on CT thorax as in chest x-ray.

**Figure 4:**
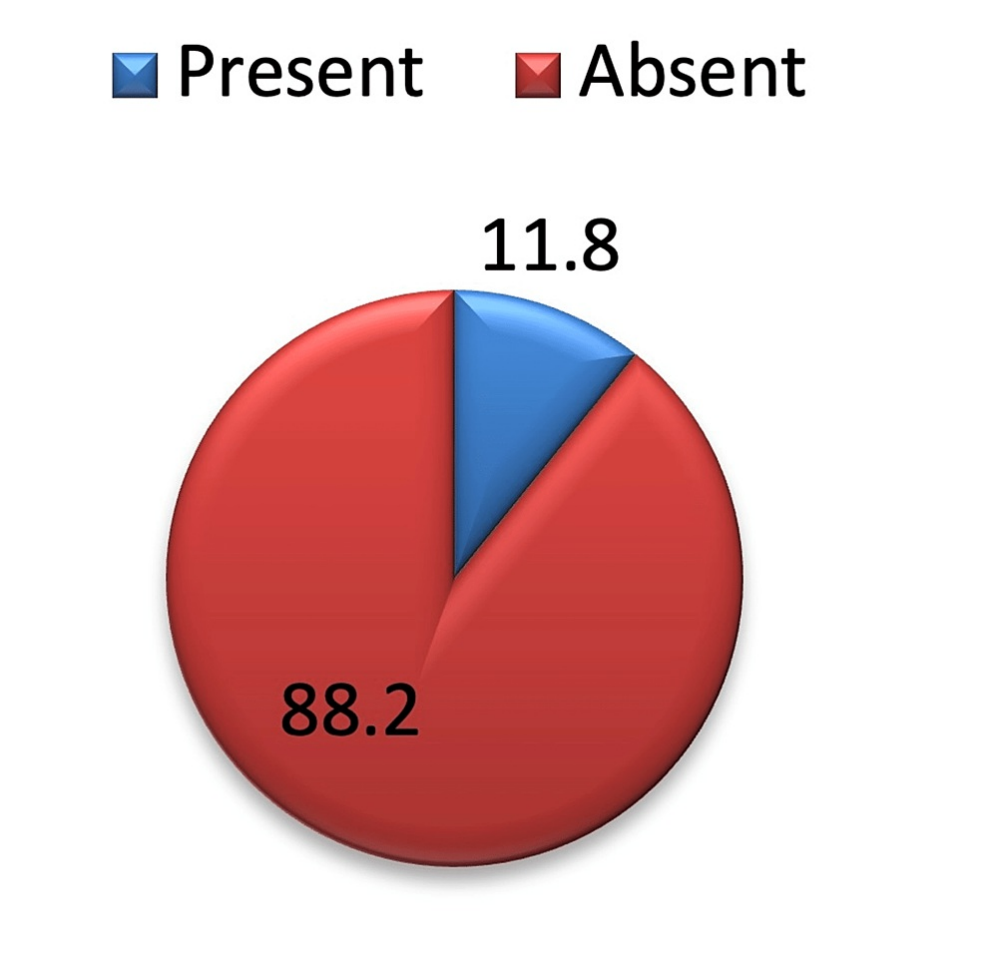
Post-intercostal tube drainage air leak

The mean chest tube duration was 4.39 ±1.18 days (Table 2). Out of 102 study subjects, 12 (11.8 %) had post-operative air leak (Figure 4). The mean hospital stay was 5.75±1.49 days (Table 3). We didn’t find any chest tube complications after the insertion of the chest tube and Thopaz^+^ application.

**Table 2:** Total duration of chest tube (in days)

Duration of Chest Tube (days)	Number of Patients	Percentage (%)
3	28	27.5
4	29	28.4
5	29	28.4
6	10	9.8
7	5	4.8
8	1	0.98
Mean (Standard Deviation)	4.39 (1.18)	

**Table 3:** Duration of hospital stay (in days)

Duration of Hospital Stay (Days)	Number of Patients	Percentage (%)
4	21	20.6
5	30	29.4
6	25	24.5
7	14	13.7
>7	12	11.9
Mean (Standard Deviation)	5.75 (1.49)	
Range	4-11	

**Figure 5:**
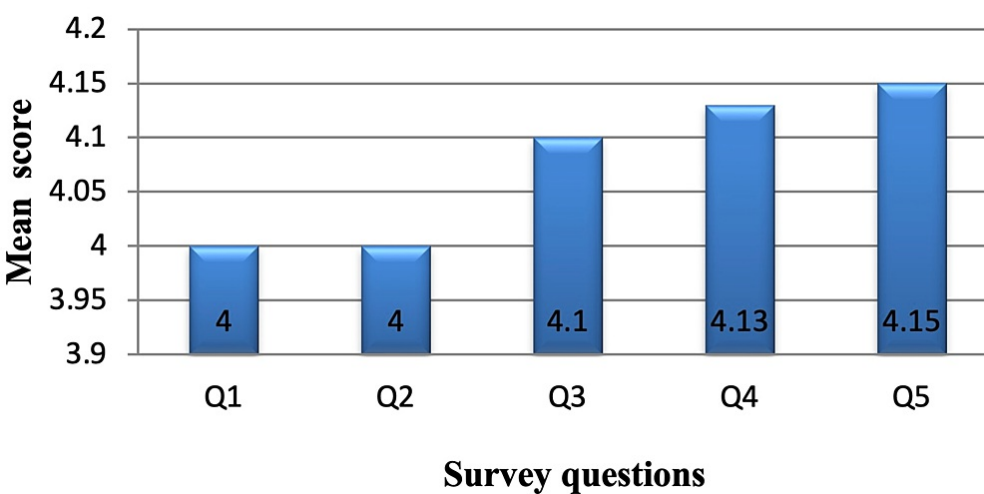
Graph showing mean scores of survey questions

Most of our patients had a positive response for the digital device with a mean score of 4 for each survey question (Figure 5). Fifty-seven (55.9%) subjects felt comfortable to move out of bed and 42 (41.2%) felt comfortable to walk around the ward with chest drainage. Eighty-four (82.4%) chest trauma patients felt that the digital chest drainage system was convenient for other patients, 84 (82.4%) felt comfortable in bed at night for changing positions, and 72 (70.6%) patients felt safe to move around with the chest drainage system.

## Discussion

Thoracic injury is one of the major causes of mortality and morbidity. The common thoracic injuries are pneumothorax, haemothorax, hemopneumothorax, pulmonary contusion, and lung laceration. The mainstay of diagnosis lies with physical examination added with plain X-ray, pleural tap, ultrasonography, and CT scan [1]. Approximately one-quarter of civilian trauma deaths are caused by thoracic trauma and many of these deaths can be prevented by prompt diagnosis and correct management. In spite of the high mortality rates, about 90% of the patients with life-threatening thoracic injuries can be managed by a simple intervention like drainage of the pleural space by tube thoracostomy [11].

This prospective observational study of one and half year duration included 102 patients with traumatic chest injuries. In our study, chest trauma was more commonly found in males than females. Most of the subjects were in the third and fourth decades of life. The mean age of traumatic injury was 42.30 ± 15.88 years. In another study by Sharma et al., out of 730 patients, a maximum of 452 patients were between 21-30 years and the next common age group was found to be 31-40 years, with 98 patients [12]. The sex ratio was 5.3:1 in our study. The male predominance of this study was consistent with the study of Majumdar et al. [13] and Morales et al. [14]. The low incidence in females may be due to more confinement to household chores and fewer outdoor activities as compared to males [1]. These are comparable to previous studies. 

Like other studies, we also found RTA to be the most common cause of injury. Industrialisation and urbanisation with flexible rules and regulations are responsible for the increased incidence of RTA-related chest injuries [3]. The second common cause of chest trauma was a fall from height followed by penetrating injury. The most common clinical presentation was chest pain followed by tachycardia, tachypnoea, and dyspnoea. In the present study, most patients developed some type of pleural collection. Most commonly, patients had pneumothorax (61.8%) followed by hemopneumothorax (36.2%). These cases were managed with an intercostal drainage tube. After the chest tube insertion, it was connected to a digital drainage system (Thopaz^+^). Various data were recorded to evaluate the efficacy of the digital drainage system. 

Over recent years, digital drainage systems have been used to quantify air leaks and it has been suggested that such devices may provide a useful adjunct to clinical management without the need for provocative clamping [15]. Several studies have shown that the use of these devices has benefits in comparison to current practice. The use of digital systems has been associated with chest drains being removed sooner (by around two days) than a conventional system [11].

In our study, the mean chest tube duration was 4.39 ±1.18 days (range of three to eight days). The majority of our patients had a chest tube for a duration of three to five days. Only five (4.8%) patients had chest tube for seven days and one (0.98 %) had it for eight days. The higher duration of chest tube was due to the presence of post-operative air leak. Out of 102 patients, 12 (11.8%) had an air leak after chest tube insertion. All our patients were stable after chest tube insertion and didn’t develop any chest complications. None of our patients required chest tube reinsertion. We observed that the mean duration of hospital stay was 5.75±1.49 days.It ranged from 4-11 days.

The systemic review and meta-analysis by Zhou et al. found that, as compared to analogue drainage systems, the digital drainage system after pulmonary surgery is associated with shorter chest tube placement duration, less air leak, less hospital stay, and lower post-operative cost [16]. However, there was no significant difference regarding the occurrence of prolonged air leak, air leak on post-operative days 1, 2, or 3, or the percentage of patients discharged with a device. This meta-analysis of various studies measured the duration of air leak with digital drainage devices. They found that a digital drainage system was associated with a shorter duration of air leak, and they didn’t find any significant difference in subgroup analysis [16].

The mean score of our survey questionnaire was 4. It represents that patients are comfortable and have a positive perception of the digital drainage system compared to the traditional drainage systems. Our results are comparable with the study done by Pompili et al. [11] They found that study subjects treated with an electronic drainage system had a more positive response related to its comfort, portability, and convenience from personnel and patients as compared to that from study subjects treated with traditional drainage system.

A pilot study by Tunnicliffe and Draper indicated that the Thopaz^+^ digital drainage system may have a useful role in pneumothorax management [17]. The system appears to be subjectively acceptable to patients and staff. Training of staff is important when using digital devices, which will be unfamiliar to most staff caring for medical patients. This small pilot study did not identify any safety concerns. 

Results from a multicenter, international, randomized controlled trial involving a total of 381 pulmonary resection procedures performed on 191 patients receiving digital drainage and 190 patients with traditional drains showed that those randomized to digital systems had a significantly shorter duration of chest drain placement (mean of 3.6 vs 4.7 days; p=0.0001) [9].

Thopaz^+^ received a positive recommendation from the National Institute for Health and Care Excellence (NICE) and should be considered for managing chest drains. Clinical evidence for Thopaz^+^ showed shorter drainage time and length of hospital stay, lower rates of chest drain re-insertion, cost saving, and higher rates of patient satisfaction when used in patients following pulmonary resection and patients with pneumothorax compared to conventional chest drainage [8].

To the best of our knowledge, our study is the first of its kind to use Thopaz^+^ in traumatic pneumothorax/hemopneumothorax. Thopaz^+^ drainage system has been used earlier in studies involving lung surgeries but not on chest trauma patients. The earlier concept of continuous low-pressure negative suction was used to treat traumatic pneumothorax by connecting the patient chest tube with wall suction and maintaining low-pressure suction.

In our study, we found that Thopaz^+^ digital drainage system is better and more efficient than the conventional system. It reduces hospital stay, post-operative air leak, and risk of infection significantly. It also provides easy monitoring of air leak and fluid loss. Most of our patients showed positive insight into the use of the digital device because of its ease of comfort and mobility. We suggest, for the management of patients with chest trauma, a digital drainage system should be used to get better outcomes.

There are limitations in our study. First, our study is a single-centre study done on a relatively small sample size. Second, we did not include a control group to compare the outcome with a classical device (traditional chest drainage system). Third, the definition of the absence of an air leak in the digital group (< 30 mL/min for more than eight hours) is based on previous studies and experience. Fourth, the intervention was not blinded to the patients and staff in the department. Most of the previous studies have been done on post-surgical cases retrospectively in small groups of patients whereas our study was conducted in traumatic pneumothorax. Reported outcomes have been found similar to the available literature. A well-designed multicenter randomised study is needed in future to examine the actual cost, safety, and clinical benefit of digital drainage devices. Larger studies would be able to conclude that this device is very helpful with post-operative chest tube drainage. 

## Conclusions

The Thopaz^+^ device was found to be safe, comfortable, and well-tolerated among patients with chest trauma. It provides an accurate assessment of air leak, chest drainage, and is more reproducible between observers. Digital drainage systems shorten hospital stays and reduce post-operative complications, thus minimising the cost of hospitalization and treatment. It favours early mobilisation and better physiotherapy. Patients who have been managed with digital drainage devices experienced more satisfaction and convenience due to ease of use and portability.
